# Assignment of Absolute Configurations of Two Promising Anti-*Helicobacter pylori* Agents from the Marine Sponge-Derived Fungus *Aspergillus niger* L14

**DOI:** 10.3390/molecules26165061

**Published:** 2021-08-20

**Authors:** Jia Liu, Ronglu Yu, Jia Jia, Wen Gu, Huawei Zhang

**Affiliations:** 1 School of Pharmaceutical Sciences, Zhejiang University of Technology, Hangzhou 310014, China; jliu3092@163.com (J.L.); yurl0024@163.com (R.Y.); 2 Jiangsu Key Laboratory of Pathogen Biology, Department of Pathogen Biology, Nanjing Medical University, Nanjing 211166, China; jiajia@njmu.edu.cn; 3 College of Chemical Engineering, Nanjing Forestry University, Nanjing 210037, China; njguwen@163.com

**Keywords:** *Aspergillus niger*, endozoic fungus, fonsecinone A, aurasperone A, anti *Helicobacter pylori*, chiral axis

## Abstract

A chemical investigation into endozoic fungus *Aspergillus niger* L14 derived from the marine sponge of *Reniera japonica* collected off Xinghai Bay (China) resulted in the isolation of two dimeric naphtho-*γ*-pyrones, fonsecinone A (**1**) and isoaurasperone A (**2**). Through a combination of ECD spectra and X-ray diffraction analysis, the chiral axes of compounds **1** and **2** were unambiguously determined as *R_α_*-configurations. Bioassay results indicated that these substances exhibited remarkably inhibitory effects on human pathogens *Helicobacter pylori* G27 and 159 with MIC values of ≤4 μg/mL, which are similar to those of the positive control, ampicillin sodium. To the best of our knowledge, this is the first report on absolute configuration of **1** and crystallographic data of **2**, as well as their potent anti-*H. pylori* activities.

## 1. Introduction

**Figure 1:**
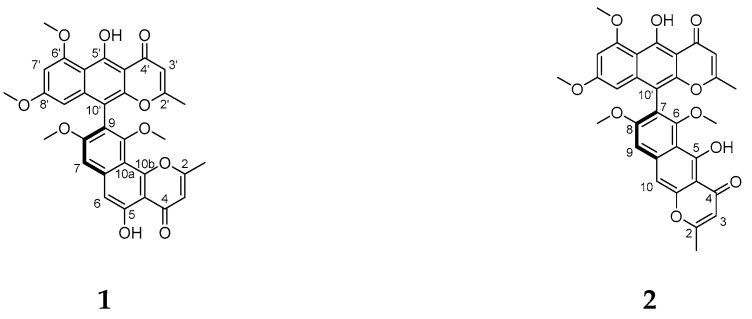
Chemical structures of compounds **1** and **2** from *Aspergillus niger* L14.

*Helicobacter pylori* is one of the most serious pathogenic bacteria that threaten human health, since it has infected approximately half of the world’s population [1]. Owing to decreasing effectiveness of existing antibiotic-based therapies, there is an urgent need to develop new antibiotics for the treatment of *H. pylori* infection [2]. As we know, most therapeutic agents currently available on the market and drug leads in clinical trial are originally derived from terrestrial microorganisms. [3] Recently, a growing body of evidence suggests that marine sponge-derived microbes are one of the more prolific sources of bioactive secondary metabolites for the development of new medicines [4,5,6,7]. Among these microorganisms, fungi in particular can produce a large number of compounds with significant bioactivities [8]. As one of the most common and important filamentous fungi in nature, *Aspergillus niger* possesses great potential to produce a remarkable array of substances of biomedical and agricultural relevance, as well as food enzymes [9,10,11]. Our previous bioassay of endozoic microbes from a specimen (MNP-2016) of *Reniera japonica* collected off Xinghai Bay (China) led to the isolation of one fungus, *A. niger* L14, with strong antimicrobial effects [12]. In order to extract novel bioactive compounds from this strain, fermentation and chemical investigation were carried out in this work. Herein, we report the isolation, structural elucidation and anti-*Helicobacter pylori* effects of two dimeric naphtho-*γ*-pyrones, namely fonsecinone A (**1**) and isoaurasperone A (**2**) (Figure 1). As a structurally unique metabolite, compound **1** was first isolated from *A. fonsecaeus* in 1984 [13], as well as from *A. aculeatus* [14], *A. fumigatus* [15] and *Pleurotus ostreatus* [16] more recently, while compound **2** was obtained from *A. niger* in 1979 for the first time and it exhibited cytotoxic and mycotoxic properties [17]. However, the compounds’ stereochemistry has not yet been determined. Therefore, the present work focus on the assignment of absolute configurations of compounds **1** and **2** by a combination of ECD spectra and single crystal X-ray diffraction analysis, as well as on the discovery of their potent anti-*H. pylori* effects.

## 2. Results and Discussion

### 2.1. Planar Structures of Compounds **1** and **2**

Through a semi-preparative HPLC technique, compounds **1** and **2** were, respectively, separated as yellow amorphous powder from an ethyl acetate extract of a solid fermented rice of strain L14. The pseudo-molecular-ion peaks at *m*/*z* 571 ([M + H]^+^) and 593 ([M + Na]^+^) in their ESI-MS spectra suggested that **1** and **2** are isomeric (Supplementary Figures S1 and S2). On basis of their similar ^1^H and ^13^C NMR spectra [18] (Supplementary Figures S3–S6), their molecular formulas are deduced as C_32_H_26_O_10_. By further careful comparison of their ^1^H and ^13^C NMR spectral data with the literature (Table 1), compounds **1** and **2** were identified as fonsecinone A [14] and isoaurasperone A [19], respectively, which are dimeric naphtho-*γ*-pyrone analogs.

### 2.2. Stereochemistry of Compounds **1** and **2**

**Figure 2:**
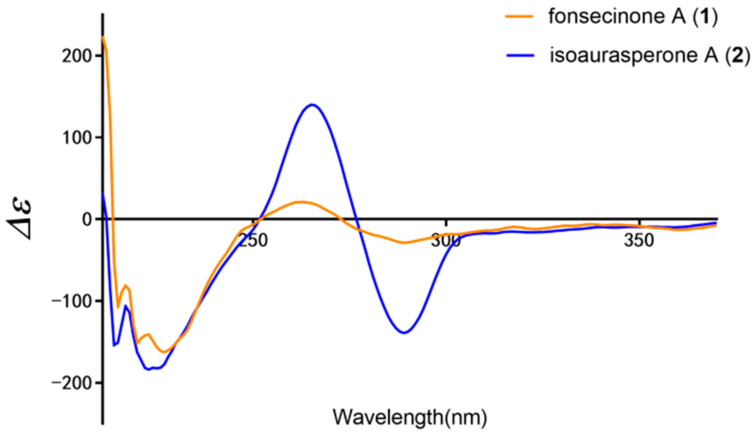
Experimental ECD spectra of compounds **1** and **2**.

**Figure 3:**
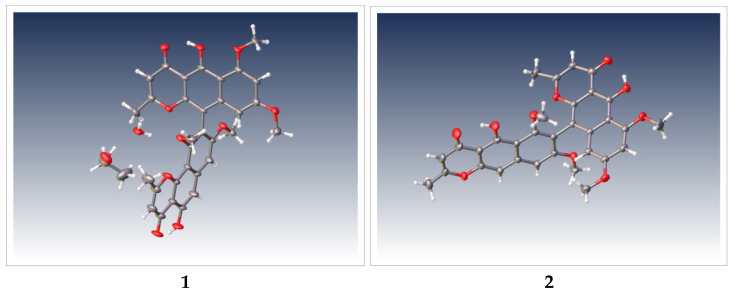
ORTEP drawings of compounds **1** and **2**.

The elucidation of stereogenicity or chirality of natural products plays a key role in the characterization of their structure features and biological properties [20]. In order to determine absolute configurations of compounds **1** and **2**, ECD and single crystal X-ray diffraction analysis were conducted. As shown in Figure 2, ECD spectra of compounds **1** and **2** had very similar cotton effects, which one valley at 280 nm and a peak at 270 nm were respectively shown in the first negative and the positive cotton effect regions while the last elliptical valley was apparent at 220 nm in the negative cotton effect region. Moreover, their ECD spectra are completely contrary to those of *S*-configured aurasperones A–C [21]. Therefore, the absolute configurations of chiral axes in compounds **1** and **2** were unambiguously identified as *R_a_*. Single crystals of **1** and **2** were successfully obtained from a mixture of chloroform and ethanol, and their crystallographic data were deposited at CCDC (Nos. 2064113 and 2064114, Figure 3). Through X-ray crystallographic analysis using Cu K_α_ radiation, the absolute configuration of chiral axes in **1** and **2** were verified as *R_a_* with a GOOF^2^ of 1.023 and 1.042, respectively. To the best of our knowledge, ours is the first report on the absolute configuration of compound **1** and the crystallographic data of compound **2**.

Crystal data for **1** is as follows: C_34_H_34_O_12_ (M_r_ = 634.61 g/mol): triclinic, space group P-1 (no. 2), *a* = 8.0417 (7) Å, *b* = 11.9593 (10) Å, *c* = 17.1410 (15) Å, *α* = 97.364 (6)°, *β* = 100.693 (6)°, *γ* = 104.037 (5)°, *V* = 1545.4 (2) Å^3^, *Z* = 2, T = 173.01 K, *μ*(GaKα) = 0.558 mm^−1^, *Dcalc* = 1.364 g/cm^3^, 15,365 reflections were measured (7.458° ≤ 2Θ ≤ 110.172°), 5733 unique (*R*_int_ = 0.0733, R_sigma_ = 0.0843), which were used in all calculations. The final *R*_1_ was 0.0956 (I > 2σ(I)) and *wR*_2_ was 0.3083 for all data. Each molecule of fonsecinone A (**1**) was co-crystallized with a water molecule and an ethanol molecule (Supplementary Tables S1–S4).

Crystal data for **2** is as follows: C_32_H_26_O_10_, (M_r_ = 570.53 g/mol), monoclinic, space group P2_1_/c (no. 14), *a* = 11.7688 (5) Å, *b* = 13.7141 (7) Å, *c* = 17.5181 (8) Å, *β* = 109.295 (3)°, *V* = 2668.6 (2) Å^3^, *Z* = 4, T = 193.0 K, *μ*(GaKα) = 0.570 mm^−1^, *Dcalc* = 1.420 g/cm^3^, 27,052 reflections were measured (6.924° ≤ 2Θ ≤ 110.078°), 5071 unique (*R*_int_ = 0.0543, *R*_sigma_ = 0.0381), which were used in all calculations. The final *R*_1_ was 0.0508 (I > 2σ(I)) and *wR*_2_ was 0.1499 for all data. (Supplementary Tables S1, S5–S7).

CCDC Nos. 2064113 and 2064114 contain the supplementary crystallographic data for this paper. These data are freely available via http://www.ccdc.cam.ac.uk/conts/retrieving.html (accessed on 21 February 2021) or can be obtained from the CCDC, 12 Union Road, Cambridge CB2 1EZ, UK; Fax: +44 1223 336033; E-mail: deposit@ccdc.cam.ac.uk)

### 2.3. Antimicrobial Activity of Compounds **1** and **2**

Bioassay results indicated that these bis-naphtho-*γ*-pyrones **1** and **2** exhibited potent antimicrobial effects on human pathogen *H. pylori* G27 with minimum inhibitory concentration (MIC) values of 2 μg/mL, as well as multi-drug resistant *H. pylori* 159 with MIC values of 2 and 4 μg/mL, respectively (Table 2). It is noteworthy that several previous reports suggested that compounds **1** and **2** had no significant bioactivity against pathogenic microbes *Alternaria solani*, *Bacillus cereus*, *B. subtilis*, *Escherichia coli*, *Pseudomonas fluorescens*, *Trichophyton rubrum* and *Candida albican*, and displayed a weak inhibitory effect on xanthine oxidase and no cytotoxicities against the following panel of human cancer cell lines: A2780, H1688, K562, M231, PC3, A549, MGC-803 and HL-60 [21,22,23,24,25,26]. Therefore, the present work provides an important discovery suggesting that compounds **1** and **2** are potential new drug candidates for the treatment of *H. pylori* infections.

### 2.4. Putative Biosynthetic Pathway of Compounds **1** and **2**

**Figure 4:**
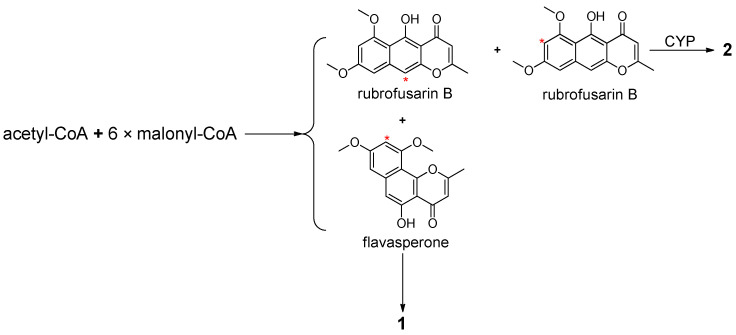
Putative biosynthetic pathways of compounds **1** and **2**.

Biosynthetically, naphtho-*γ*-pyrone monomers rubrofusarin B and flavasperone are aromatic polyketides, which are formed by one acetyl-CoA and six malonyl-CoA through assembly reaction catalyzed by polyketide synthase (PKS), followed by condensation, cyclization, dehydration and methylation [15,27]. Compound **2** is the dimerization product of two molecules of rubrofusarin B at C-7 and C-10, respectively, and it has been shown to be catalyzed by the pre-anthraquinone-dimerizing cytochrome P450 (CYP) enzyme encoded by the gene *aunB* [28] (Figure 4). Therefore, it is inferred that compound **1** is the dimerization product of rubrofusarin B and flavasperone at C-10 and C-9, respectively.

## 3. Materials and Methods

### 3.1. General Experimental Procedures

NMR spectra were collected by Bruker Avance DRX600 spectrometer (Bruker, Fällande, Switzerland) equipped with a 5 mm triple resonance (HCN) cold probe, using TMS as an internal standard. ESI-MS and HR-ESI-MS data were obtained from an Agilent 6210 LC/TOF-MS spectrometer (Agilent Technologies, Santa Clara, CA, USA). A suitable crystal was selected and measured on a Bruker APEX-II CCD diffractometer (Bruker, Fällande, Switzerland). The structure was solved with the ShelXT structure solution program using intrinsic phasing and refined with the ShelXL refinement package using least squares minimization [29]. A high performance liquid chromatography (HPLC) system, Essentia LC-16P apparatus (Shimadzu, Kyoto, Japan), equipped with a semi-preparative column (Phenomenex Hydro-RP, 250 mm × 10 mm, 4 µm, Torrance, CA, USA), was used to purify all compounds. Acetonitrile (Merck, Darmstadt, Germany) and H_2_O used in HPLC system were chromatographic grade, and all other chemicals were analytical grade.

### 3.2. Fungal Strain

Endozoic strain L14 was isolated from fresh specimens (MNP-2016) of *Reniera japonica* collected at Xinghai Bay (Dalian, China), as described in the literature [11], and identified as *A. niger* according to its morphological characteristics and molecular phylogeny based on 18*S* rDNA gene sequence (GenBank accession No. MF093522).

### 3.3. Fermentation and Extraction

First, strain L14 was cultured on potato dextrose agar (PDA) at 30 °C for 5 days. Then, a balanced amount of fungal colony was transferred to the culture broth in a 500 mL Erlenmeyer flask containing 300 mL sterilized potato dextrose broth (PDB); it was then shaken at 180 rpm for 3 days under 30 °C as seed broth. After that, the seed broth was inoculated to a solid rice medium in a 1000 mL Erlenmeyer flask with sterilized rice (160 g) and water (320 mL) and cultivated at 20 °C for 40 days. At the end of fermentation, all fermented material was collected and extracted with the same volume of ethyl acetate 3 times. The organic layer was concentrated under vacuum at 38 °C to obtain crude extract (approx. 50 g).

### 3.4. Isolation and Purification

The ethyl acetate extract was separated into six fractions, A-F, under a gradient condition of CH_3_CN and H_2_O on a preparative HPLC column, according to our previous established method [30]. Fraction D was further subjected to HPLC fractionation to generate fonsecinone A (**1**) (159.4 mg, t_R_ = 22.6 min) and isoaurasperone A (**2**) (156.3 mg, t_R_ = 24.3 min) using a semi-preparative C_18_ column under a gradient condition of mobile phase (CH_3_CN and H_2_O) with a flow rate of 3.0 mL/min.

### 3.5. Antimicrobial Assay

Human pathogen stain *H. pylori* G27 was a clinically susceptible strain, while multi-drug resistant clinical strain *H. pylori* 159 was obtained from a biopsy sample of a gastritis patient. Isolation and identification of *H. pylori* 159 were used standard protocols on the basis of colony appearance, Gram staining and positive reactions in the rapid urease test [31]. Then, 10% fetal calf serum (FCS) brain heart infusion (BHI) (Becton Dickinson, Sparks Glencoe, MD, USA) broth or 5% FCS Columbia blood agar (Oxoid, Basingstoke, UK), supplemented with Dent selective supplement (Oxoid, Basingstoke, UK), was used for routine culture of *H. pylori* strains. Incubation of strains was conducted under microaerophilic conditions (10% CO_2_, 85% N_2_, and 5% O_2_ and 90% relative humidity) using a double-gas CO_2_ incubator (Binder, model CB160, Germany) at 37 °C for 48 to 72 h. Three replicates were performed for every antimicrobial assay.

Anti-*H. pylori* activities were carried out according to broth microdilution assay [32]. Briefly, two-fold serial dilutions of compounds were prepared in a 96-well microtiter plate containing 100 μL of BHI broth containing 10% FCS. An overnight *H.* *pylori* liquid culture was diluted in BHI broth and was inoculated into each well with a final concentration of 5 × 10^5^ CFU/mL. After being incubated in a microaerophilic atmosphere at 37 °C for 2 days, the plates were examined visually. The MIC was determined to be the lowest concentration with no turbidity in well. For quality control and comparative analysis, the conventional antibiotics metronidazole (MTZ), clarithromycin (CLR) and levofloxacin (LVX) were tested as positive control. All MIC assays were performed in triplicate at least. Antimicrobial activity testing of each compound followed antimicrobial susceptibility testing standards outlined by the document M07-A7 (Clinical and Laboratory Standards Institute 2008) against strains G27 and Hp159.

## Figures and Tables

**Table 1:** ^13^C and ^1^H NMR spectral data for compounds **1** and **2**.

	Compound	1		2	
Position		δ_C_^a^	δ_H_^b^	δ_C_^a^	δ_H_^b^
2		167.6		167.7	
3		110.8	6.32 (1H, s)	107.6	6.06 (1H, s)
4		183.1		184.7	
4a		109.5		104.9	
5		156.8		162.1	
5a		/		111.6	
6		106.2	7.04 (1H, s)	158.7	
6a		140.9		/	
7		101.7	6.97 (1H, s)	117.8	
8		160.2		160.3	
9		117.3		101.5	6.97 (1H, s)
9a		/		140.8	
10		157.1		101.4	7.16 (1H, s)
10a		108.1		153.5	
10 b		155.2		/	
2-Me		20.7	2.48 (3H, s)	21	2.42 (3H, s)
5-OH			12.82 (1H, s)		
6-OMe		/		62.2	3.46 (3H, s)
8-OMe		56.1	3.78 (3H, s)	56.1	3.79 (3H, s)
			3.43 (3H, s)		
10-OMe		61.2	3.78 (3H, s)	/	3.43 (3H, s)
			3.43 (3H, s)		
2′		167		167.8	
3′		107.5	5.99 (1H, s)	107.4	5.98 (1H, s)
4′		184.7		184.6	
4′a		104.4		104.4	
5′		163		162.9	
5′a		108.8		108.7	
6′		161.3		161.2	
7′		97.1	6.42 (1H, d)	97	6.42 (1H, s)
8′		161.8		161.5	
9′		96.5	6.19 (1H, d)	96.7	6.21 (1H, s)
9′a		140.8		140.7	
10′		105.2		105.3	
10′a		151		151	
2′-Me		20.8	2.12 (3H, s)	20.8	2.12 (3H, s)
5′-OH			15.23 (1H, s)		
6′-OMe		56.4	4.02 (3H, s)	56.4	4.03 (3H, s)
8′-OMe		55.3	3.61 (3H, s)	55.3	3.62 (3H, s)

^a^: in CDCl_3_, 150 MHz; ^b^: in CDCl_3_, 600 MHz.

**Table 2:** In vitro anti-*Helicobacter pylori* effects of compounds **1** and **2**.

Compound	MIC Value (μg/mL)	
	Strain G27 (S)	Strain 159 (R)
**1**	2	2
**2**	2	4
MTZ	2	16
CLR	0.004	2
LVX	0.5	8

Note: Antimicrobial susceptibility breakpoints were determined according to EUCAST (European Committee on Antimicrobial Susceptibility Testing) guidelines. S, drug susceptible; R, drug resistant; MTZ, metronidazole; CLR, clarithromycin; LVX, levofloxacin.

## Data Availability

Not applicable.

## Supplementary Materials

The following are available online, Figure S1: ESI-MS spectrum of compound **1**, Figure S2: ESI-MS spectrum of **2**, Figure S3: ^1^H NMR spectrum (600 MHz, CDCl_3_) of **1**, Figure S4: ^13^C NMR spectrum (150 MHz, CDCl_3_) of **1**, Figure S5: ^1^H NMR spectrum (600 MHz, CDCl_3_) of **2**, Figure S6: ^13^C NMR spectrum (150 MHz, CDCl_3_) of **2**, Figure S7: Crystallographic shape of **1** and **2**, Table S1: Selected crystal data and refinement parameters for **1** and **2**, Table S2: Atomic coordinates (× 10^4^) and equivalent isotropic displacement parameters (Å^2^ × 10^3^) for **1**, Table S3: Bond lengths [Å] and angles [°] for **1**, Table S4: Hydrogen coordinates (× 10^4^) and isotropic displacement parameters (Å^2^ × 10^3^) for **1**, Table S5: Atomic coordinates (× 10^4^) and equivalent isotropic displacement parameters (Å^2^ × 10^3^) for **2**, Table S6: Bond lengths [Å] and angles [°] for **2**, Table S7: Hydrogen coordinates (× 10^4^) and isotropic displacement parameters (Å^2^ × 10^3^) for **2**.

## Sample Availability

Strain L14 and compounds **1** and **2** are available from the authors.
