# Supplementary material for: Assignment of Absolute Configurations of Two Promising Anti-Helicobacter pylori Agents from the Marine Sponge-Derived Fungus Aspergillus niger L14
Source: Molecules. 2021 Aug 20;26(16):5061. doi: 10.3390/molecules26165061 (PMC8399357; doi:10.3390/molecules26165061)
Supplement: Supplementary file 1 [file molecules-26-05061-s001.zip › molecules-1322550-SI.pdf]

Supplementary materials for

Assignment of absolute configurations of two promising anti-*Helicobacter pylori* agents from the marine sponge-derived fungus *Aspergillus niger* L14

Jia Liu, Ronglu Yu, Jia Jia, Wen Gu and Huawei Zhang *

* Correspondence:: hwzhang@zjut.edu.cn; Tel.: +86-571-8832-0913

Contents

Figure S1. ESI-MS spectrum of compound **1**..3

Figure S2. ESI-MS spectrum of compound **2**..4

Figure S3. ^{1}H NMR spectrum (600 MHz, $CDCl_3$) of compound **1**..5

Figure S4. ^{13}C NMR spectrum (150 MHz, $CDCl_3$) of compound **1**..6

Figure S5. ^{1}H NMR spectrum (600 MHz, $CDCl_3$) of compound **2**..7

Figure S6. ^{13}C NMR spectrum (150 MHz, $CDCl_3$) of compound **2**..8

Figure S7. Crystal shapes of compounds **1** and **2**..9

Table S1. Selected crystallographic data and refinement parameters for compounds **1** and **2**..10

Table S2. Atomic coordinates (x 10^4) and equivalent isotropic displacement parameters ($Å^2$ x 10^3) for compound **1**……………………………………….11

Table S3. Bond lengths [Å] and angles [°] for compound **1**…………………………………………………………………………......................……12

Table S4. Hydrogen coordinates (x 10^4) and isotropic displacement parameters ($Å^2$ x 10^3) for compound **1**……………………………..…15

Table S5. Atomic coordinates (x 10^4) and equivalent isotropic displacement parameters ($Å^2$ x 10^3) for compound **2**…………………………………………16

Table S6. Bond lengths [Å] and angles [°] for compound **2**……………………………………………………………………………………...…17

Table S7. Hydrogen coordinates (x 10^4) and isotropic displacement parameters ($Å^2$ x 10^3) for compound **2**……………………………………..….……20

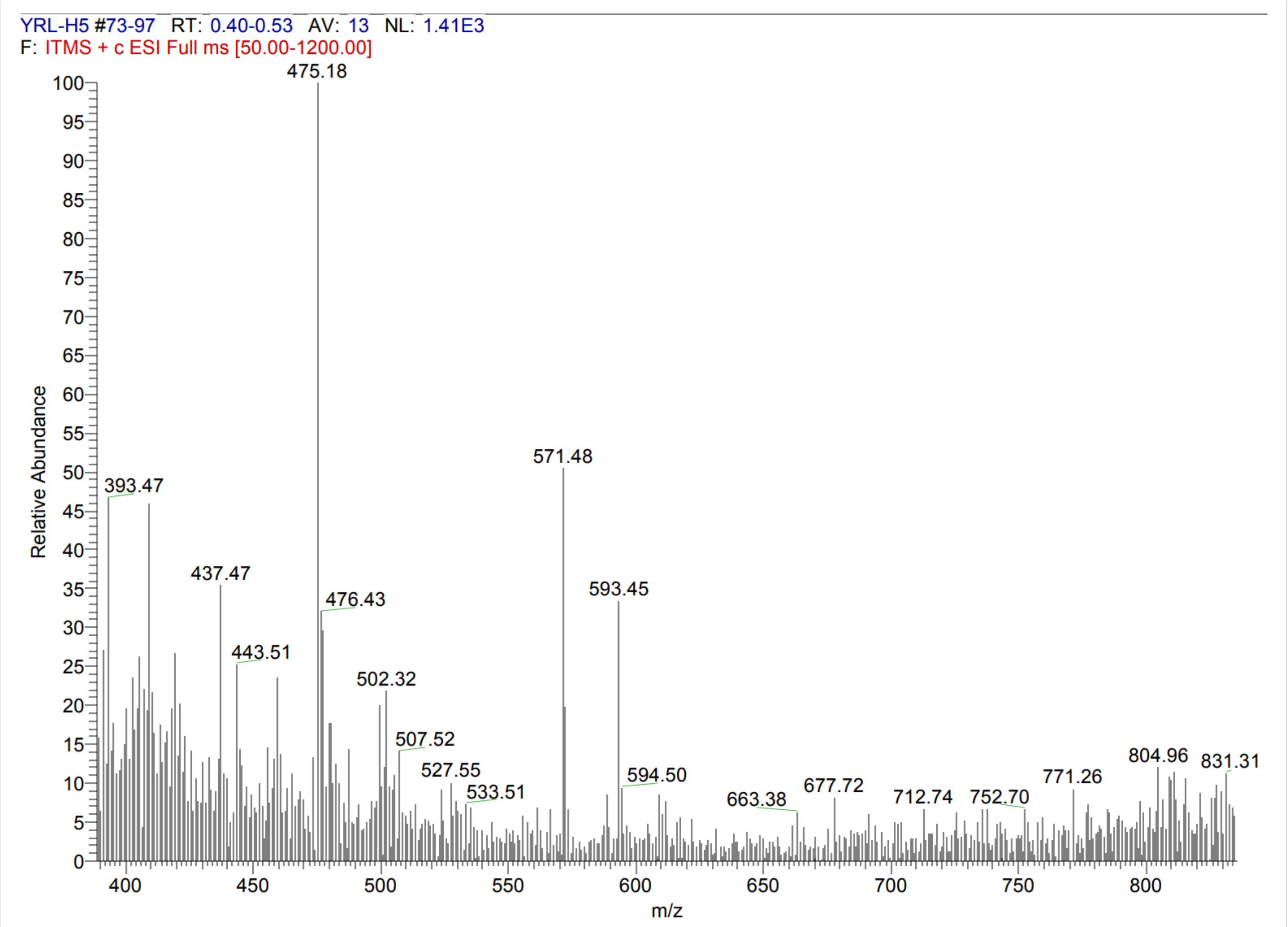

Figure S1. ESI-MS spectrum of compound **1.**

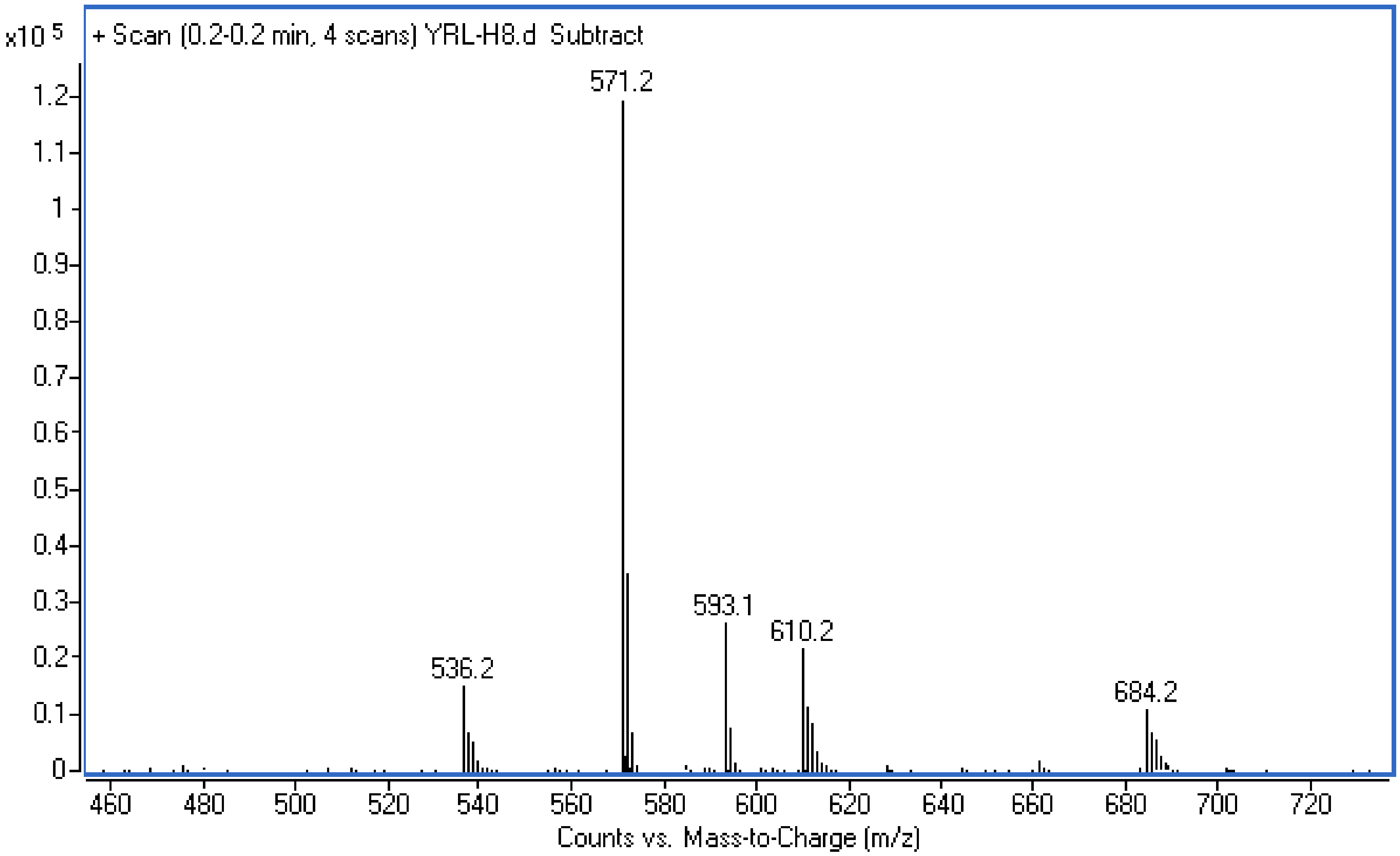

Figure S2. ESI-MS spectrum of compound **2.**

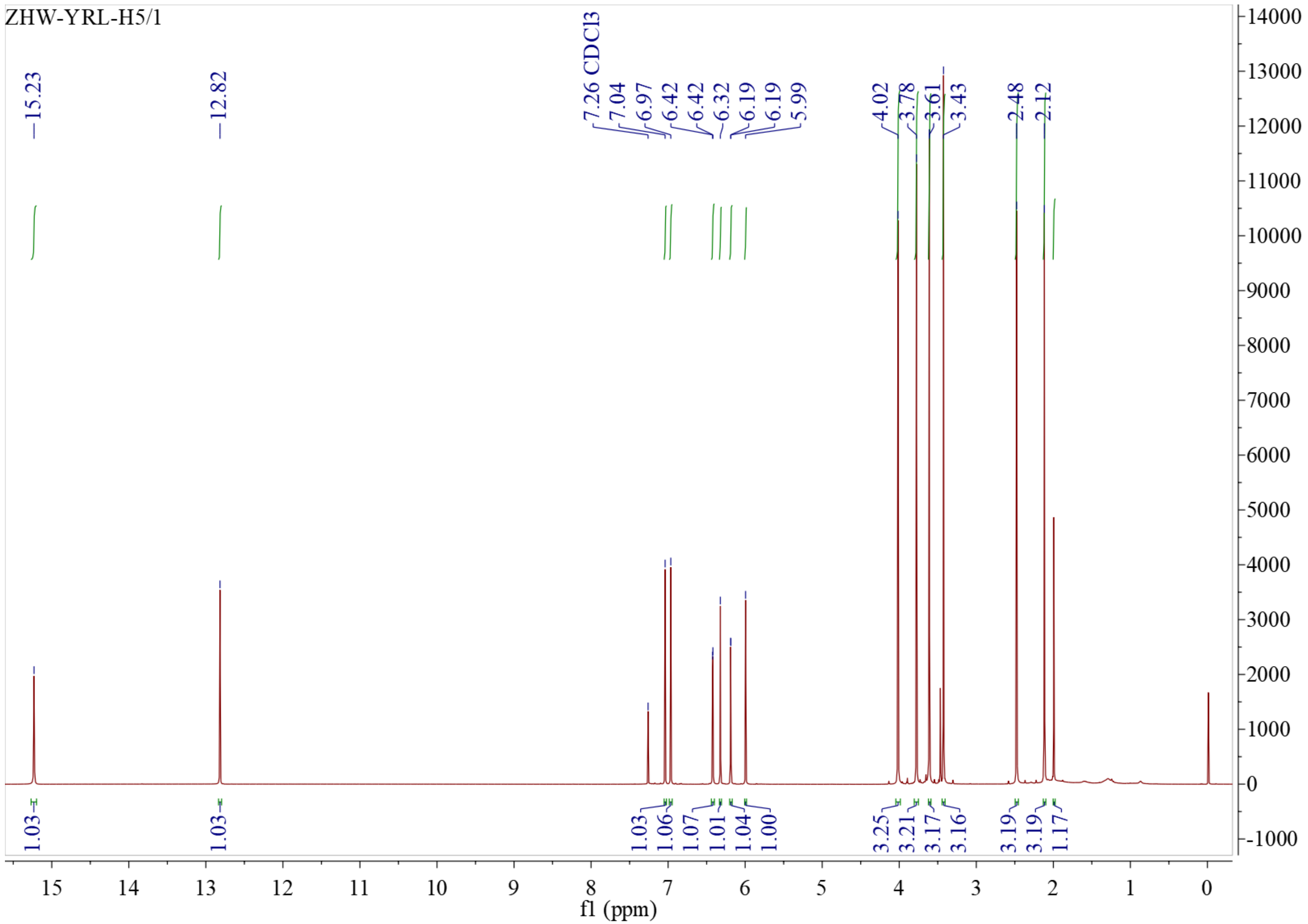

Figure S3. ^{1}H NMR spectrum (600 MHz, $CDCl_3$) of compound **1.**

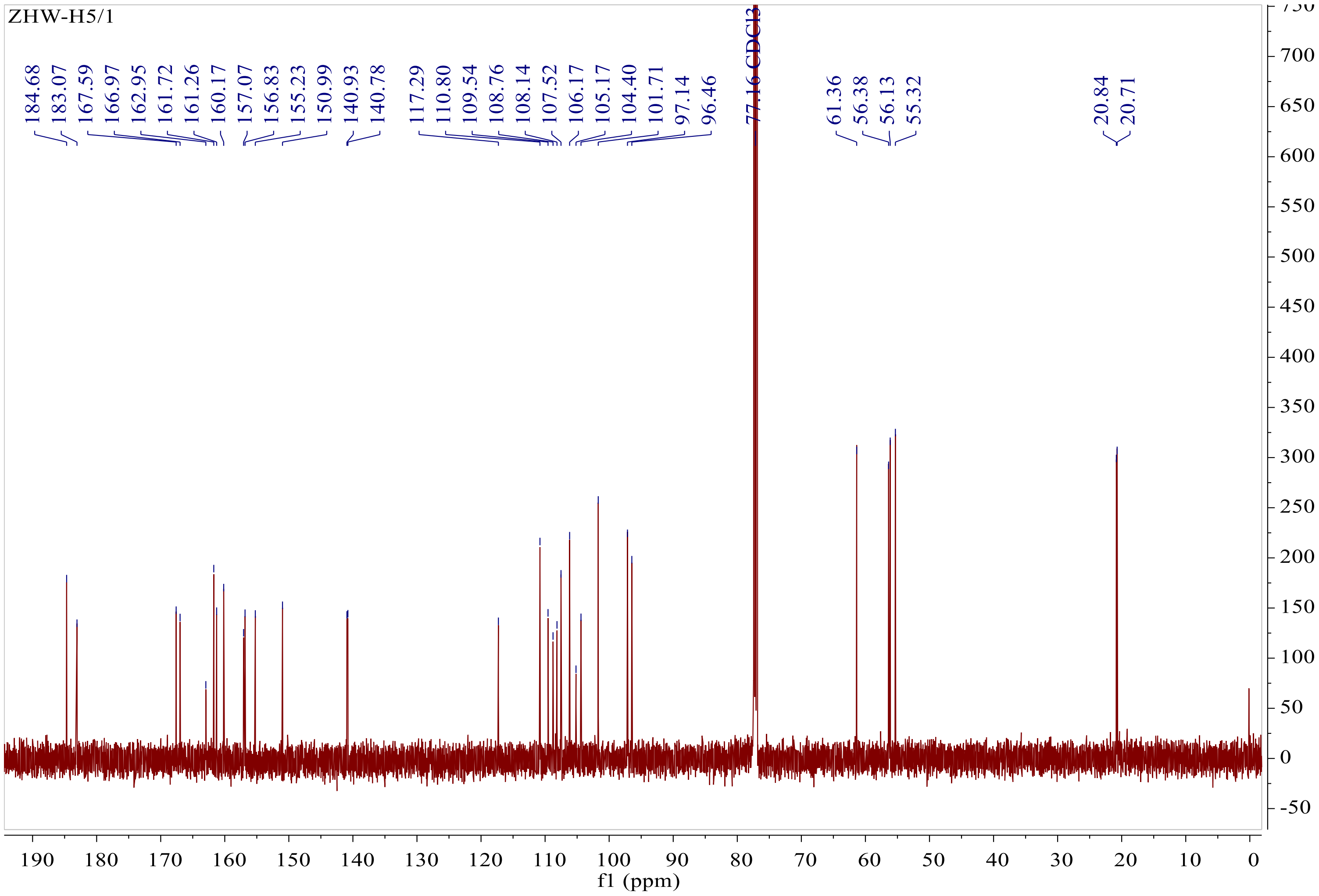

Figure S4. ^{13}C NMR spectrum (150 MHz, $CDCl_3$) of compound **1.**

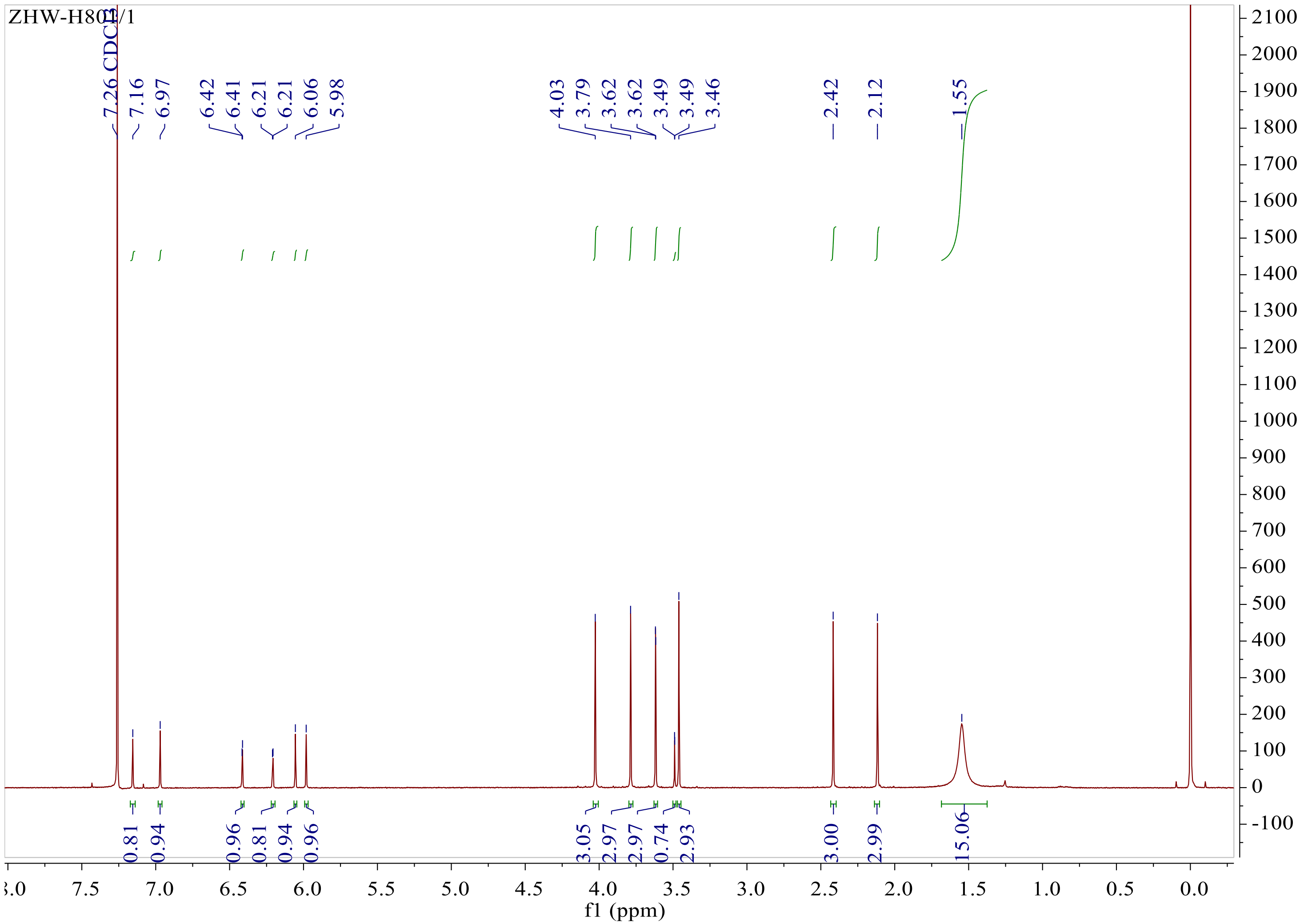

Figure S5. ^{1}H NMR spectrum (600 MHz, $CDCl_3$) of compound **2.**

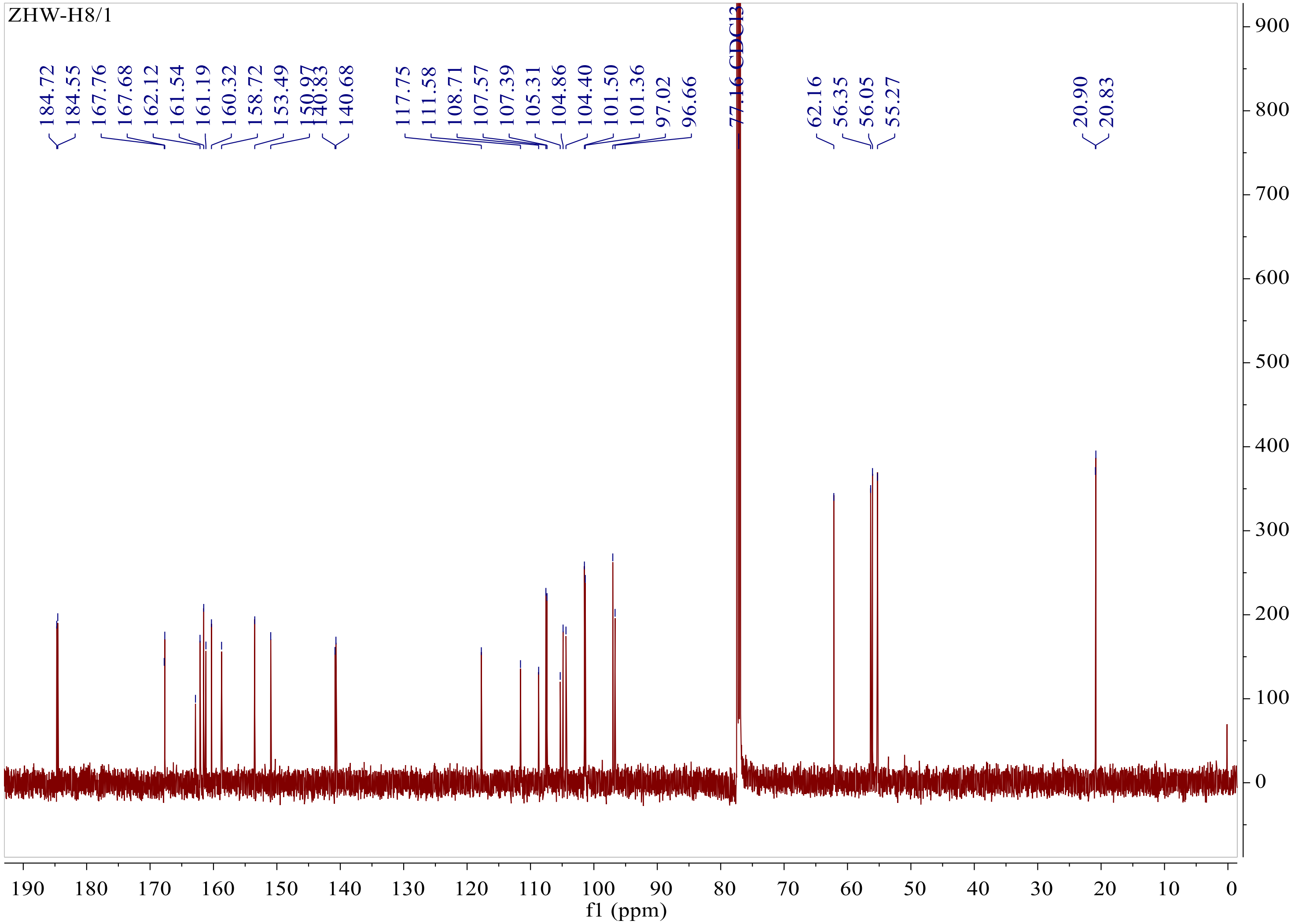

Figure S6. ^{13}C NMR spectrum (150 MHz, $CDCl_3$) of compound **2.**

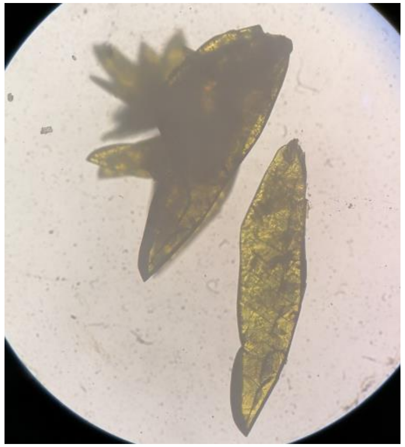

1

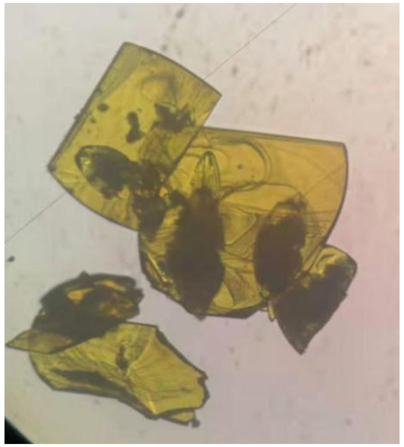

2

Figure S7. Crystal shapes of compounds **1** and **2.**

Table S1. Selected crystallographic data and refinement parameters for compounds **1** and **2**.

Compound / parameters	**1**	**2**
Formula weight	634.61	570.53
Temperature (K)	173.01	193
Crystal color	yellow	yellow
Crystal size (mm^3)	0.05 × 0.03 × 0.01	0.05 × 0.05 × 0.01
Crystal system	triclinic	monoclinic
Space group	P-1	$P2_1/c$
a/Å	8.0417(7)	11.7688(5)
b/Å	11.9593(10)	13.7141(7)
c/Å	17.1410(15)	17.5181(8)
α/°	97.364(6)	90
β/°	100.693(6)	109.295(3)
γ/°	104.037(5)	90
V (Å^3)	1545.4	2668.6
Z	2	4
ρ_{calc}(g/cm^3)	1.364	1.420
μ/mm^{-1}	0.558	0.570
F(000)	668.0	1192.0
Radiation	GaKα (λ = 1.34139)	GaKα (λ = 1.34139)
Range of h, k and l	−9 ⩽ h ⩽ 9, −13 ⩽ k ⩽ 14, −20 ⩽ l ⩽ 20	−14 ⩽ h ⩽ 9, −16 ⩽ k ⩽ 16, −21 ⩽ l ⩽ 20
Goodness-of-fit on F^2	1.023	1.042
Final R indexes [I>=2σ (I)]	R_1 = 0.0956, wR_2 = 0.2537	R_1 = 0.0508, wR_2 = 0.1356
Final R indexes [all data]	R_1 = 0.1535, wR_2 = 0.3083	R_1 = 0.0669, wR_2 = 0.1499

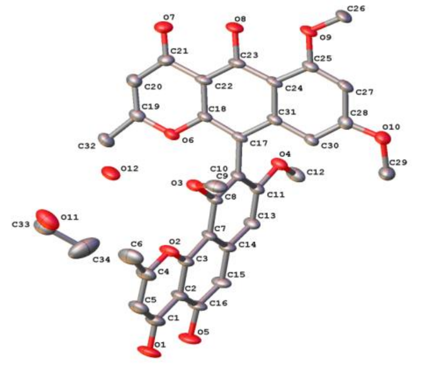

Table S2. Atomic coordinates (× 10^4) and equivalent isotropic displacement parameters (Å^2 × 10^3) for compound **1**.

	x	*y*	*z*	U(eq)*
O(1)	2566(5)	2228(3)	3812(2)	84(1)
O(2)	6316(4)	3297(3)	5896(1)	58(1)
O(3)	7445(3)	4461(2)	7387(1)	45(1)
O(4)	4744(3)	7329(2)	8231(1)	47(1)
O(5)	971(4)	3651(3)	4318(2)	75(1)
O(6)	5612(3)	4349(2)	8784(1)	47(1)
O(7)	8398(4)	4720(3)	11090(2)	61(1)
O(8)	10342(4)	6633(3)	10944(1)	55(1)
O(9)	12163(3)	8614(2)	10735(1)	53(1)
O(10)	10497(4)	10071(3)	8375(2)	60(1)
C(1)	3737(6)	2557(4)	4460(2)	66(1)
C(2)	3661(6)	3471(4)	5086(2)	54(1)
C(3)	4985(5)	3814(4)	5792(2)	47(1)
C(4)	6395(7)	2429(4)	5315(2)	68(1)
C(5)	5157(7)	2054(5)	4620(3)	72(1)
C(6)	7956(8)	1989(6)	5567(3)	94(2)
C(7)	4974(5)	4703(3)	6431(2)	43(1)
C(8)	6198(4)	5048(3)	7192(2)	41(1)
C(9)	9114(5)	4950(5)	7219(2)	63(1)
C(10)	6102(4)	5918(3)	7783(2)	41(1)
C(11)	4739(5)	6485(3)	7623(2)	42(1)
C(12)	3313(6)	7840(4)	8129(3)	62(1)
C(13)	3531(5)	6161(3)	6896(2)	48(1)
C(14)	3608(5)	5258(4)	6299(2)	48(1)
C(15)	2275(5)	4910(4)	5572(2)	57(1)
C(16)	2288(5)	4029(4)	4994(2)	56(1)
C(17)	7278(4)	6165(3)	8604(2)	41(1)
C(18)	6977(4)	5345(3)	9099(2)	40(1)
C(19)	5153(5)	3531(4)	9256(2)	50(1)
C(20)	6042(5)	3641(4)	10009(2)	51(1)
C(21)	7516(5)	4611(4)	10384(2)	49(1)
C(22)	8009(5)	5506(4)	9890(2)	45(1)
C(23)	9357(5)	6504(3)	10184(2)	45(1)
C(24)	9749(4)	7411(3)	9720(2)	43(1)
C(25)	11090(5)	8475(3)	9990(2)	44(1)
C(26)	13619(5)	9633(4)	10976(2)	58(1)
C(27)	11298(5)	9322(4)	9524(2)	49(1)
C(28)	10180(5)	9134(3)	8750(2)	46(1)
C(29)	9281(6)	10017(4)	7642(3)	64(1)
C(30)	8910(5)	8105(3)	8432(2)	43(1)
C(31)	8633(4)	7219(3)	8916(2)	40(1)
C(32)	3619(5)	2557(4)	8790(2)	57(1)
O(11)	4640(6)	125(4)	6722(3)	105(1)
C(33)	2902(7)	134(5)	6720(3)	82(2)
C(34)	2412(10)	1152(9)	6280(4)	142(3)

O(12)	6899(5)	2151(3)	7672(2)	77(1)

*: U(eq) is defined as one third of the trace of the orthogonalized U^{ij} tensor.

Table S3. Bond lengths [Å] and angles [°] for compound **1**.

Bond lengths [Å]		Bond angles [°]	
O(1)-C(1)	1.263(4)	C(3)-O(2)-C(4)	121.0(3)
O(2)-C(3)	1.355(4)	C(8)-O(3)-C(9)	115.5(3)
O(2)-C(4)	1.365(5)	C(11)-O(4)-C(12)	117.1(3)
O(3)-C(8)	1.374(4)	C(16)-O(5)-H(5)	109.5
O(3)-C(9)	1.426(5)	C(19)-O(6)-C(18)	120.2(3)
O(4)-C(11)	1.353(4)	C(23)-O(8)-H(8)	109.5
O(4)-C(12)	1.423(4)	C(25)-O(9)-C(26)	117.0(3)
O(5)-H(5)	0.8400	C(28)-O(10)-C(29)	116.9(3)
O(5)-C(16)	1.358(4)	O(1)-C(1)-C(2)	120.8(4)
O(6)-C(18)	1.377(4)	O(1)-C(1)-C(5)	122.4(4)
O(6)-C(19)	1.373(5)	C(5)-C(1)-C(2)	116.8(3)
O(7)-C(21)	1.258(4)	C(3)-C(2)-C(1)	119.0(4)
O(8)-H(8)	0.8400	C(3)-C(2)-C(16)	119.4(4)
O(8)-C(23)	1.361(4)	C(16)-C(2)-C(1)	121.6(3)
O(9)-C(25)	1.368(4)	O(2)-C(3)-C(2)	120.6(3)
O(9)-C(26)	1.424(5)	O(2)-C(3)-C(7)	117.9(3)
O(10)-C(28)	1.357(5)	C(2)-C(3)-C(7)	121.5(3)
O(10)-C(29)	1.425(4)	O(2)-C(4)-C(6)	111.2(3)
C(1)-C(2)	1.451(6)	C(5)-C(4)-O(2)	121.4(4)
C(1)-C(5)	1.415(6)	C(5)-C(4)-C(6)	127.4(4)
C(2)-C(3)	1.396(4)	C(1)-C(5)-H(5A)	119.4
C(2)-C(16)	1.418(6)	C(4)-C(5)-C(1)	121.2(4)
C(3)-C(7)	1.428(5)	C(4)-C(5)-H(5A)	119.4
C(4)-C(5)	1.348(6)	C(4)-C(6)-H(6A)	109.5
C(4)-C(6)	1.489(6)	C(4)-C(6)-H(6B)	109.5
C(5)-H(5A)	0.9500	C(4)-C(6)-H(6C)	109.5
C(6)-H(6A)	0.9800	H(6A)-C(6)-H(6B)	109.5
C(6)-H(6B)	0.9800	H(6A)-C(6)-H(6C)	109.5
C(6)-H(6C)	0.9800	H(6B)-C(6)-H(6C)	109.5
C(7)-C(8)	1.424(4)	C(8)-C(7)-C(3)	125.2(3)
C(7)-C(14)	1.413(5)	C(14)-C(7)-C(3)	116.9(3)
C(8)-C(10)	1.383(5)	C(14)-C(7)-C(8)	117.9(3)
C(9)-H(9A)	0.9800	O(3)-C(8)-C(7)	120.6(3)
C(9)-H(9B)	0.9800	O(3)-C(8)-C(10)	117.3(3)
C(9)-H(9C)	0.9800	C(10)-C(8)-C(7)	121.9(3)
C(10)-C(11)	1.426(5)	O(3)-C(9)-H(9A)	109.5
C(10)-C(17)	1.491(4)	O(3)-C(9)-H(9B)	109.5
C(11)-C(13)	1.375(4)	O(3)-C(9)-H(9C)	109.5
C(12)-H(12A)	0.9800	H(9A)-C(9)-H(9B)	109.5
C(12)-H(12B)	0.9800	H(9A)-C(9)-H(9C)	109.5
C(12)-H(12C)	0.9800	H(9B)-C(9)-H(9C)	109.5
C(13)-H(13)	0.9500	C(8)-C(10)-C(11)	118.8(3)
C(13)-C(14)	1.411(5)	C(8)-C(10)-C(17)	120.4(3)
C(14)-C(15)	1.426(4)	C(11)-C(10)-C(17)	120.5(3)
C(15)-H(15)	0.9500	O(4)-C(11)-C(10)	115.1(3)
C(15)-C(16)	1.354(6)	O(4)-C(11)-C(13)	124.5(3)
C(17)-C(18)	1.384(5)	C(13)-C(11)-C(10)	120.5(3)
C(17)-C(31)	1.417(5)	O(4)-C(12)-H(12A)	109.5
C(18)-C(22)	1.414(4)	O(4)-C(12)-H(12B)	109.5
C(19)-C(20)	1.328(5)	O(4)-C(12)-H(12C)	109.5
C(19)-C(32)	1.489(6)	H(12A)-C(12)-H(12B)	109.5
C(20)-H(20)	0.9500	H(12A)-C(12)-H(12C)	109.5
C(20)-C(21)	1.422(6)	H(12B)-C(12)-H(12C)	109.5
C(21)-C(22)	1.475(6)	C(11)-C(13)-H(13)	119.6
C(22)-C(23)	1.367(6)	C(11)-C(13)-C(14)	120.7(3)

C(23)-C(24)	1.435(6)
C(24)-C(25)	1.413(5)
C(24)-C(31)	1.455(4)
C(25)-C(27)	1.366(6)
C(26)-H(26A)	0.9800
C(26)-H(26B)	0.9800
C(26)-H(26C)	0.9800
C(27)-H(27)	0.9500
C(27)-C(28)	1.414(5)
C(28)-C(30)	1.366(5)
C(29)-H(29A)	0.9800
C(29)-H(29B)	0.9800
C(29)-H(29C)	0.9800
C(30)-H(30)	0.9500
C(30)-C(31)	1.428(5)
C(32)-H(32A)	0.9800
C(32)-H(32B)	0.9800
C(32)-H(32C)	0.9800
O(11)-H(11)	0.8400
O(11)-C(33)	1.400(7)
C(33)-H(33A)	0.9900
C(33)-H(33B)	0.9900
C(33)-C(34)	1.601(9)
C(34)-H(34A)	0.9800
C(34)-H(34B)	0.9800
C(34)-H(34C)	0.9800
O(12)-H(12D)	0.8698
O(12)-H(12E)	0.8698

C(14)-C(13)-H(13)	119.6
C(7)-C(14)-C(15)	121.1(3)
C(13)-C(14)-C(7)	120.1(3)
C(13)-C(14)-C(15)	118.8(3)
C(14)-C(15)-H(15)	119.9
C(16)-C(15)-C(14)	120.2(3)
C(16)-C(15)-H(15)	119.9
O(5)-C(16)-C(2)	118.6(4)
C(15)-C(16)-O(5)	120.6(4)
C(15)-C(16)-C(2)	120.8(3)
C(18)-C(17)-C(10)	118.3(3)
C(18)-C(17)-C(31)	118.3(3)
C(31)-C(17)-C(10)	123.4(3)
O(6)-C(18)-C(17)	116.6(3)
O(6)-C(18)-C(22)	121.0(3)
C(17)-C(18)-C(22)	122.4(3)
O(6)-C(19)-C(32)	110.1(3)
C(20)-C(19)-O(6)	121.9(4)
C(20)-C(19)-C(32)	128.0(4)
C(19)-C(20)-H(20)	118.7
C(19)-C(20)-C(21)	122.6(4)
C(21)-C(20)-H(20)	118.7
O(7)-C(21)-C(20)	123.7(4)
O(7)-C(21)-C(22)	120.2(4)
C(20)-C(21)-C(22)	116.2(3)
C(18)-C(22)-C(21)	118.0(3)
C(23)-C(22)-C(18)	119.6(4)
C(23)-C(22)-C(21)	122.3(3)
O(8)-C(23)-C(22)	118.5(4)
O(8)-C(23)-C(24)	119.6(3)
C(22)-C(23)-C(24)	122.0(3)
C(23)-C(24)-C(31)	116.6(3)
C(25)-C(24)-C(23)	125.3(3)
C(25)-C(24)-C(31)	118.1(4)
O(9)-C(25)-C(24)	116.9(3)
C(27)-C(25)-O(9)	121.9(3)
C(27)-C(25)-C(24)	121.2(3)
O(9)-C(26)-H(26A)	109.5
O(9)-C(26)-H(26B)	109.5
O(9)-C(26)-H(26C)	109.5
H(26A)-C(26)-H(26B)	109.5
H(26A)-C(26)-H(26C)	109.5
H(26B)-C(26)-H(26C)	109.5
C(25)-C(27)-H(27)	120.0
C(25)-C(27)-C(28)	120.1(4)
C(28)-C(27)-H(27)	120.0
O(10)-C(28)-C(27)	113.3(3)
O(10)-C(28)-C(30)	124.7(3)
C(30)-C(28)-C(27)	122.1(4)
O(10)-C(29)-H(29A)	109.5
O(10)-C(29)-H(29B)	109.5
O(10)-C(29)-H(29C)	109.5
H(29A)-C(29)-H(29B)	109.5
H(29A)-C(29)-H(29C)	109.5
H(29B)-C(29)-H(29C)	109.5
C(28)-C(30)-H(30)	120.6
C(28)-C(30)-C(31)	118.9(3)
C(31)-C(30)-H(30)	120.6
C(17)-C(31)-C(24)	121.1(3)
C(17)-C(31)-C(30)	119.4(3)
C(30)-C(31)-C(24)	119.6(3)
C(19)-C(32)-H(32A)	109.5
C(19)-C(32)-H(32B)	109.5

C(19)-C(32)-H(32C)	109.5
H(32A)-C(32)-H(32B)	109.5
H(32A)-C(32)-H(32C)	109.5
H(32B)-C(32)-H(32C)	109.5
C(33)-O(11)-H(11)	109.5
O(11)-C(33)-H(33A)	109.5
O(11)-C(33)-H(33B)	109.5
O(11)-C(33)-C(34)	110.5(5)
H(33A)-C(33)-H(33B)	108.1
C(34)-C(33)-H(33A)	109.5
C(34)-C(33)-H(33B)	109.5
C(33)-C(34)-H(34A)	109.5
C(33)-C(34)-H(34B)	109.5
C(33)-C(34)-H(34C)	109.5
H(34A)-C(34)-H(34B)	109.5
H(34A)-C(34)-H(34C)	109.5
H(34B)-C(34)-H(34C)	109.5
H(12D)-O(12)-H(12E)	104.5

Table S4. Hydrogen coordinates (× 10^4) and isotropic displacement parameters (Å^2 × 10^3) for compound **1**.

	x	*y*	*z*	U(eq)
H(5)	1124	3073	4035	113
H(8)	9953	6050	11149	83
H(5A)	5239	1440	4230	86
H(6A)	9032	2631	5653	141
H(6B)	7967	1358	5144	141
H(6C)	7897	1689	6070	141
H(9A)	8974	4941	6638	94
H(9B)	9919	4486	7392	94
H(9C)	9597	5760	7512	94
H(12A)	3392	8313	7704	93
H(12B)	3360	8341	8636	93
H(12C)	2201	7220	7976	93
H(13)	2634	6549	6795	57
H(15)	1373	5298	5492	68
H(20)	5679	3048	10309	61
H(26A)	14373	9640	10587	88
H(26B)	14296	9627	11512	88
H(26C)	13190	10334	10993	88
H(27)	12194	10038	9720	59
H(29A)	9364	9403	7226	96
H(29B)	9559	10774	7464	96
H(29C)	8084	9839	7732	96
H(30)	8223	7981	7898	51
H(32A)	2655	2878	8572	85
H(32B)	3232	2017	9145	85
H(32C)	3961	2135	8344	85
H(11)	5331	682	7070	158
H(33A)	2755	260	7282	98
H(33B)	2094	-634	6436	98
H(34A)	1459	804	5800	212
H(34B)	3447	1597	6119	212
H(34C)	2028	1678	6651	212
H(12D)	6786	2809	7535	116
H(12E)	8026	2271	7845	116

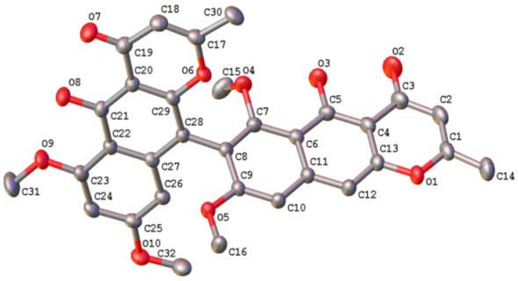

Table S5. Atomic coordinates (× 10^4) and equivalent isotropic displacement parameters ($Å^2$ × 10^3) for compound **2**.

	x	*y*	*z*	U(eq)
O(1)	1514(1)	7943(1)	−296(1)	52(1)
O(2)	-321(2)	6261(2)	815(1)	83(1)
O(3)	1416(1)	6393(1)	2125(1)	65(1)
O(4)	3128(1)	6861(1)	3492(1)	56(1)
O(5)	6528(1)	8549(1)	3352(1)	44(1)
O(6)	3979(1)	8727(1)	4380(1)	44(1)
O(7)	5557(2)	9065(1)	6811(1)	62(1)
O(8)	7156(1)	7837(1)	6781(1)	54(1)
O(9)	8624(1)	6512(1)	6671(1)	59(1)
O(10)	8338(1)	5189(1)	4119(1)	57(1)
C(1)	385(2)	7615(2)	−702(1)	52(1)
C(2)	−235(2)	7064(2)	−355(1)	61(1)
C(3)	242(2)	6783(2)	480(1)	58(1)
C(4)	1433(2)	7153(1)	919(1)	47(1)
C(5)	1990(2)	6960(1)	1746(1)	46(1)
C(6)	3139(2)	7355(1)	2169(1)	42(1)
C(7)	3741(2)	7262(1)	3024(1)	42(1)
C(8)	4857(2)	7654(1)	3394(1)	39(1)
C(9)	5431(2)	8178(1)	2922(1)	39(1)
C(10)	4883(2)	8306(1)	2111(1)	42(1)
C(11)	3733(2)	7904(1)	1719(1)	41(1)
C(12)	3166(2)	8077(1)	883(1)	45(1)
C(13)	2045(2)	7720(1)	511(1)	44(1)
C(14)	−19(2)	7974(2)	−1548(1)	69(1)
C(15)	3484(3)	5912(2)	3799(1)	67(1)
C(16)	7068(2)	9215(2)	2944(1)	51(1)
C(17)	3504(2)	9330(1)	4808(1)	49(1)
C(18)	4008(2)	9465(1)	5601(1)	52(1)
C(19)	5072(2)	8951(1)	6063(1)	48(1)
C(20)	5565(2)	8283(1)	5609(1)	40(1)
C(21)	6606(2)	7734(1)	5982(1)	42(1)
C(22)	7067(2)	7088(1)	5523(1)	40(1)
C(23)	8095(2)	6466(1)	5851(1)	46(1)
C(24)	8489(2)	5866(1)	5373(1)	49(1)
C(25)	7872(2)	5835(1)	4531(1)	46(1)
C(26)	6894(2)	6409(1)	4182(1)	42(1)
C(27)	6473(2)	7048(1)	4667(1)	38(1)
C(28)	5439(2)	7627(1)	4290(1)	38(1)
C(29)	5007(2)	8204(1)	4769(1)	38(1)
C(30)	2375(2)	9784(2)	4273(2)	63(1)
C(31)	9640(2)	5911(2)	7031(2)	86(1)
C(32)	7686(2)	5087(2)	3277(1)	63(1)

Table S6. Bond lengths [Å] and angles [°] for compound **2**.

Bond lengths [Å]		Bond angles [°]	
O(1)-C(1)	1.360(2)	C(1)-O(1)-C(13)	119.73(17)
O(1)-C(13)	1.379(2)	C(5)-O(3)-H(3)	109.5
O(2)-C(3)	1.246(3)	C(7)-O(4)-C(15)	116.47(16)
O(3)-H(3)	0.8400	C(9)-O(5)-C(16)	117.28(14)
O(3)-C(5)	1.340(2)	C(17)-O(6)-C(29)	119.98(15)
O(4)-C(7)	1.374(2)	C(21)-O(8)-H(8)	109.5
O(4)-C(15)	1.419(3)	C(23)-O(9)-C(31)	117.71(18)
O(5)-C(9)	1.360(2)	C(25)-O(10)-C(32)	115.92(16)
O(5)-C(16)	1.432(2)	O(1)-C(1)-C(14)	110.4(2)
O(6)-C(17)	1.356(2)	C(2)-C(1)-O(1)	122.6(2)
O(6)-C(29)	1.377(2)	C(2)-C(1)-C(14)	127.1(2)
O(7)-C(19)	1.254(2)	C(1)-C(2)-H(2)	119.0
O(8)-H(8)	0.8400	C(1)-C(2)-C(3)	122.01(19)
O(8)-C(21)	1.341(2)	C(3)-C(2)-H(2)	119.0
O(9)-C(23)	1.365(2)	O(2)-C(3)-C(2)	123.0(2)
O(9)-C(31)	1.417(3)	O(2)-C(3)-C(4)	121.5(2)
O(10)-C(25)	1.367(2)	C(2)-C(3)-C(4)	115.5(2)
O(10)-C(32)	1.427(3)	C(5)-C(4)-C(3)	121.37(19)
C(1)-C(2)	1.330(3)	C(5)-C(4)-C(13)	119.13(17)
C(1)-C(14)	1.482(3)	C(13)-C(4)-C(3)	119.50(19)
C(2)-H(2)	0.9500	O(3)-C(5)-C(4)	118.78(17)
C(2)-C(3)	1.435(3)	O(3)-C(5)-C(6)	120.80(18)
C(3)-C(4)	1.450(3)	C(4)-C(5)-C(6)	120.43(17)
C(4)-C(5)	1.404(3)	C(5)-C(6)-C(7)	124.63(17)
C(4)-C(13)	1.405(3)	C(5)-C(6)-C(11)	117.98(17)
C(5)-C(6)	1.418(3)	C(11)-C(6)-C(7)	117.38(16)
C(6)-C(7)	1.435(3)	O(4)-C(7)-C(6)	118.88(16)
C(6)-C(11)	1.429(2)	C(8)-C(7)-O(4)	119.08(16)
C(7)-C(8)	1.367(3)	C(8)-C(7)-C(6)	121.68(16)
C(8)-C(9)	1.424(2)	C(7)-C(8)-C(9)	119.29(16)
C(8)-C(28)	1.492(2)	C(7)-C(8)-C(28)	121.59(16)
C(9)-C(10)	1.363(2)	C(9)-C(8)-C(28)	118.86(16)
C(10)-H(10)	0.9500	O(5)-C(9)-C(8)	114.41(15)
C(10)-C(11)	1.412(3)	O(5)-C(9)-C(10)	124.42(16)
C(11)-C(12)	1.415(3)	C(10)-C(9)-C(8)	121.15(17)
C(12)-H(12)	0.9500	C(9)-C(10)-H(10)	119.8
C(12)-C(13)	1.355(3)	C(9)-C(10)-C(11)	120.31(17)
C(14)-H(14A)	0.9800	C(11)-C(10)-H(10)	119.8
C(14)-H(14B)	0.9800	C(10)-C(11)-C(6)	120.15(16)
C(14)-H(14C)	0.9800	C(10)-C(11)-C(12)	119.13(16)
C(15)-H(15A)	0.9800	C(12)-C(11)-C(6)	120.70(17)
C(15)-H(15B)	0.9800	C(11)-C(12)-H(12)	120.4
C(15)-H(15C)	0.9800	C(13)-C(12)-C(11)	119.21(18)
C(16)-H(16A)	0.9800	C(13)-C(12)-H(12)	120.4
C(16)-H(16B)	0.9800	O(1)-C(13)-C(4)	120.68(17)
C(16)-H(16C)	0.9800	C(12)-C(13)-O(1)	116.86(17)
C(17)-C(18)	1.330(3)	C(12)-C(13)-C(4)	122.46(18)
C(17)-C(30)	1.486(3)	C(1)-C(14)-H(14A)	109.5
C(18)-H(18)	0.9500	C(1)-C(14)-H(14B)	109.5
C(18)-C(19)	1.432(3)	C(1)-C(14)-H(14C)	109.5
C(19)-C(20)	1.455(3)	H(14A)-C(14)-H(14B)	109.5
C(20)-C(21)	1.401(3)	H(14A)-C(14)-H(14C)	109.5
C(20)-C(29)	1.404(3)	H(14B)-C(14)-H(14C)	109.5
C(21)-C(22)	1.420(3)	O(4)-C(15)-H(15A)	109.5
C(22)-C(23)	1.436(3)	O(4)-C(15)-H(15B)	109.5
C(22)-C(27)	1.432(2)	O(4)-C(15)-H(15C)	109.5
C(23)-C(24)	1.360(3)	H(15A)-C(15)-H(15B)	109.5
C(24)-H(24)	0.9500	H(15A)-C(15)-H(15C)	109.5
C(24)-C(25)	1.413(3)	H(15B)-C(15)-H(15C)	109.5
C(25)-C(26)	1.362(3)	O(5)-C(16)-H(16A)	109.5

C(26)-H(26)	0.9500
C(26)-C(27)	1.419(2)
C(27)-C(28)	1.420(2)
C(28)-C(29)	1.366(2)
C(30)-H(30A)	0.9800
C(30)-H(30B)	0.9800
C(30)-H(30C)	0.9800
C(31)-H(31A)	0.9800
C(31)-H(31B)	0.9800
C(31)-H(31C)	0.9800
C(32)-H(32A)	0.9800
C(32)-H(32B)	0.9800
C(32)-H(32C)	0.9800

O(5)-C(16)-H(16B)	109.5
O(5)-C(16)-H(16C)	109.5
H(16A)-C(16)-H(16B)	109.5
H(16A)-C(16)-H(16C)	109.5
H(16B)-C(16)-H(16C)	109.5
O(6)-C(17)-C(30)	110.67(18)
C(18)-C(17)-O(6)	122.62(18)
C(18)-C(17)-C(30)	126.71(19)
C(17)-C(18)-H(18)	119.1
C(17)-C(18)-C(19)	121.83(18)
C(19)-C(18)-H(18)	119.1
O(7)-C(19)-C(18)	122.77(18)
O(7)-C(19)-C(20)	121.59(18)
C(18)-C(19)-C(20)	115.64(17)
C(21)-C(20)-C(19)	121.90(17)
C(21)-C(20)-C(29)	118.79(16)
C(29)-C(20)-C(19)	119.28(17)
O(8)-C(21)-C(20)	118.42(16)
O(8)-C(21)-C(22)	120.93(16)
C(20)-C(21)-C(22)	120.66(17)
C(21)-C(22)-C(23)	124.97(17)
C(21)-C(22)-C(27)	118.14(16)
C(27)-C(22)-C(23)	116.89(16)
O(9)-C(23)-C(22)	115.14(17)
C(24)-C(23)-O(9)	123.12(17)
C(24)-C(23)-C(22)	121.73(18)
C(23)-C(24)-H(24)	120.0
C(23)-C(24)-C(25)	119.95(18)
C(25)-C(24)-H(24)	120.0
O(10)-C(25)-C(24)	114.43(17)
C(26)-C(25)-O(10)	124.33(18)
C(26)-C(25)-C(24)	121.23(17)
C(25)-C(26)-H(26)	120.1
C(25)-C(26)-C(27)	119.87(18)
C(27)-C(26)-H(26)	120.1
C(26)-C(27)-C(22)	120.32(16)
C(26)-C(27)-C(28)	118.87(16)
C(28)-C(27)-C(22)	120.80(16)
C(27)-C(28)-C(8)	121.81(16)
C(29)-C(28)-C(8)	119.79(15)
C(29)-C(28)-C(27)	118.34(16)
O(6)-C(29)-C(20)	120.59(15)
C(28)-C(29)-O(6)	116.23(16)
C(28)-C(29)-C(20)	123.18(16)
C(17)-C(30)-H(30A)	109.5
C(17)-C(30)-H(30B)	109.5
C(17)-C(30)-H(30C)	109.5
H(30A)-C(30)-H(30B)	109.5
H(30A)-C(30)-H(30C)	109.5
H(30B)-C(30)-H(30C)	109.5
O(9)-C(31)-H(31A)	109.5
O(9)-C(31)-H(31B)	109.5
O(9)-C(31)-H(31C)	109.5
H(31A)-C(31)-H(31B)	109.5
H(31A)-C(31)-H(31C)	109.5
H(31B)-C(31)-H(31C)	109.5
O(10)-C(32)-H(32A)	109.5
O(10)-C(32)-H(32B)	109.5
O(10)-C(32)-H(32C)	109.5
H(32A)-C(32)-H(32B)	109.5
H(32A)-C(32)-H(32C)	109.5
H(32B)-C(32)-H(32C)	109.5
C(20)-C(21)-C(22)	120.66(17)

C(21)-C(22)-C(23)	124.97(17)
C(21)-C(22)-C(27)	118.14(16)
C(27)-C(22)-C(23)	116.89(16)
O(9)-C(23)-C(22)	115.14(17)
C(24)-C(23)-O(9)	123.12(17)
C(24)-C(23)-C(22)	121.73(18)
C(23)-C(24)-H(24)	120.0
C(23)-C(24)-C(25)	119.95(18)
C(25)-C(24)-H(24)	120.0
O(10)-C(25)-C(24)	114.43(17)
C(26)-C(25)-O(10)	124.33(18)
C(26)-C(25)-C(24)	121.23(17)
C(25)-C(26)-H(26)	120.1
C(25)-C(26)-C(27)	119.87(18)
C(27)-C(26)-H(26)	120.1
C(26)-C(27)-C(22)	120.32(16)
C(26)-C(27)-C(28)	118.87(16)
C(28)-C(27)-C(22)	120.80(16)
C(27)-C(28)-C(8)	121.81(16)
C(29)-C(28)-C(8)	119.79(15)
C(29)-C(28)-C(27)	118.34(16)
O(6)-C(29)-C(20)	120.59(15)
C(28)-C(29)-O(6)	116.23(16)
C(28)-C(29)-C(20)	123.18(16)
C(17)-C(30)-H(30A)	109.5
C(17)-C(30)-H(30B)	109.5
C(17)-C(30)-H(30C)	109.5
H(30A)-C(30)-H(30B)	109.5
H(30A)-C(30)-H(30C)	109.5
H(30B)-C(30)-H(30C)	109.5
O(9)-C(31)-H(31A)	109.5
O(9)-C(31)-H(31B)	109.5
O(9)-C(31)-H(31C)	109.5
H(31A)-C(31)-H(31B)	109.5
H(31A)-C(31)-H(31C)	109.5
H(31B)-C(31)-H(31C)	109.5
O(10)-C(32)-H(32A)	109.5
O(10)-C(32)-H(32B)	109.5
O(10)-C(32)-H(32C)	109.5
H(32A)-C(32)-H(32B)	109.5
H(32A)-C(32)-H(32C)	109.5
H(32B)-C(32)-H(32C)	109.5

Table S7. Hydrogen coordinates (× 10^4) and isotropic displacement parameters (Å^2 × 10^3) for compound **2**.

	x	*y*	*z*	U(eq)
H(3)	745	6227	1798	97
H(8)	6758	8222	6968	81
H(2)	−1017	6849	-669	74
H(10)	5277	8666	1809	51
H(12)	3566	8439	585	54
H(14A)	−145	8681	−1551	104
H(14B)	−776	7653	−1855	104
H(14C)	595	7826	−1795	104
H(15A)	3193	5434	3361	101
H(15B)	3141	5764	4226	101
H(15C)	4364	5880	4023	101
H(16A)	7229	8878	2497	76
H(16B)	7825	9461	3324	76
H(16C)	6519	9762	2730	76
H(18)	3653	9915	5867	62
H(24)	9178	5468	5605	59
H(26)	6493	6381	3614	50
H(30A)	2522	10081	3805	95
H(30B)	2109	10286	4574	95
H(30C)	1750	9284	4086	95
H(31A)	10293	6100	6829	129
H(31B)	9424	5228	6891	129
H(31C)	9907	5990	7619	129
H(32A)	7795	5674	2990	94
H(32B)	6829	4998	3199	94
H(32C)	7987	4519	3064	94
